# Integrating Stomach Content and Stable Isotope Analyses to Quantify the Diets of Pygoscelid Penguins

**DOI:** 10.1371/journal.pone.0026642

**Published:** 2011-10-28

**Authors:** Michael J. Polito, Wayne Z. Trivelpiece, Nina J. Karnovsky, Elizabeth Ng, William P. Patterson, Steven D. Emslie

**Affiliations:** 1 Department of Biology and Marine Biology, University of North Carolina Wilmington, Wilmington, North Carolina, United States of America; 2 Antarctic Ecosystem Research Division, National Marine Fisheries Service, Southwest Fisheries Science Center, La Jolla, California, United States of America; 3 Department of Biology, Pomona College, Claremont, California, United States of America; 4 Isotope Laboratory, Department of Geological Sciences, Saskatchewan, University of Saskatchewan, Saskatoon, Saskatchewan, Canada; Phillip Island Nature Parks, Australia

## Abstract

Stomach content analysis (SCA) and more recently stable isotope analysis (SIA) integrated with isotopic mixing models have become common methods for dietary studies and provide insight into the foraging ecology of seabirds. However, both methods have drawbacks and biases that may result in difficulties in quantifying inter-annual and species-specific differences in diets. We used these two methods to simultaneously quantify the chick-rearing diet of Chinstrap (*Pygoscelis antarctica*) and Gentoo (*P. papua*) penguins and highlight methods of integrating SCA data to increase accuracy of diet composition estimates using SIA. SCA biomass estimates were highly variable and underestimated the importance of soft-bodied prey such as fish. Two-source, isotopic mixing model predictions were less variable and identified inter-annual and species-specific differences in the relative amounts of fish and krill in penguin diets not readily apparent using SCA. In contrast, multi-source isotopic mixing models had difficulty estimating the dietary contribution of fish species occupying similar trophic levels without refinement using SCA-derived otolith data. Overall, our ability to track inter-annual and species-specific differences in penguin diets using SIA was enhanced by integrating SCA data to isotopic mixing modes in three ways: 1) selecting appropriate prey sources, 2) weighting combinations of isotopically similar prey in two-source mixing models and 3) refining predicted contributions of isotopically similar prey in multi-source models.

## Introduction

Stomach content analysis (SCA) is one of the most common methods for dietary analysis and provides insight into the foraging ecology of seabirds and the distribution, abundance and demography of their prey [1], [2]. Early studies often involved sacrificing animals to examine stomach contents [3], while currently a non-destructive, but still invasive, “lavage” technique to force regurgitation is commonly applied [4], [5]. When recovered stomach contents are relatively undigested, it is possible to estimate the composition and frequency occurrence of prey species and often measure, weigh and sex individual prey [3]. In addition, identifying and measuring hard prey remains, such as squid beaks and otoliths, can provide information on the size and mass of prey species when prey has been partially or completely digested [6], [7], [8].

There are inherent drawbacks and biases when using SCA to quantify seabird diets. This technique has been most commonly used during chick rearing when adults bring food ashore for their chicks; thus, less is known about the diets of seabirds outside of the breeding season [2]. Stomach contents also reflect a “snapshot” of an individual's recent diet (8–16 hours) and can be highly variable, requiring large sample sizes to statistically examine differences among species, regions and/or time [3], [9], [10]. In addition, SCA is biased towards recent dietary items and prey that does not readily digest, such as zooplankton, and can underestimate the amount of soft-bodied prey, such as fish and squid [11], [12]. While hard prey remains from stomach contents or pellets provide information on prey species composition these data are often difficult to integrate into overall diet composition estimates [6], [8], [13].

Recent advances in stable isotope analysis (SIA) and isotopic mixing models have shown great promise in quantifying the dietary composition of seabirds [14], [15]. Isotopic analyses are based on the concept that animals “are what they eat” with tissue stable nitrogen (δ^15^N) and carbon (δ^13^C) ratios reflecting diet at the time of synthesis [16]. For example, feathers are metabolically inert after synthesis, so feathers from fledgling-aged chicks integrate dietary history during the chick-rearing period as feathers replace natal down [17], [18], [19]. Isotopic mixing models use geometric or Bayesian procedures to reconstruct animal diets based on the δ^13^C and δ^15^N values of consumer tissues and isotopically distinct food sources [20], [21]. SIA and isotopic mixing models have the potential to provide relatively non-invasive and cost-effective quantitative estimates of seabird diets throughout much of their annual cycle [22], [23], [24].

There are limitations to using SIA to quantify seabird diets. When the isotopic signatures of prey species that occupy a similar trophic level overlap, such as in forage fish, overlap, it can be difficult to estimate their relative contributions to consumer diets [25], [26]. Isotopic mixing models are only as useful as the data that go into them, requiring a prior understanding of possible prey sources and their distinctive isotopic values [15]. In many cases, prior information is lacking and all possible prey sources cannot be readily identified [24]. When all prey isotopic values are not available, “representative” species are often used or multiple sources are combined *a priori* for each trophic or functional group [24], [27], [28]. Furthermore, while studies of seabird diets using SIA are becoming commonplace, few studies have compared concurrent quantitative estimates of diet composition between SCA and SIA [28], [29]. In addition, it is also common to compare SIA data to SCA prey frequency of occurrence instead of more appropriate mass-based estimates of diet composition derived from SCA [30], [31], [32].

In this study we simultaneously quantify the chick-rearing diet composition of sympatrically breeding seabirds, the Chinstrap (*Pygoscelis antarctica*) and Gentoo penguin (*P. papua*) over two breeding seasons at Cape Shirreff, Livingston Island, Antarctica (62°28′S, 60°46′W) using both SIA and SCA. Similar to other Antarctic seabirds, *Pygoscelis* penguin diets are generally composed of zooplankton, primarily Antarctic krill (*Euphausia superba*), and soft-bodied, higher-trophic prey species, such as fish [33]. As chick-rearing diets have been well studied using SCA at this site, it provides an excellent case study for comparison with SIA [34], [35], [36]. We seek to better understand the relative merits of both methods and highlight the use of SCA to inform isotopic mixing models to better quantify the diets of seabirds using SIA.

Our primary objectives are to: 1) use simultaneous collection of SCA and SIA to compare the ability of these two methods to detect inter-annual and inter-specific differences in diet composition in *Pygoscelis* penguin chicks, 2) compare the predictive ability of a two-source (krill vs. fish) linear mixing model among those using a representative fish species and those using an *a priori* averaged species and year-specific fish values, and 3) evaluate a method of *a posteriori* integrating SCA data to better elucidate the taxonomic composition of the fish portion of diets using a multi-source Bayesian mixing model.

## Materials and Methods

### Ethics statement

Animal use in this study was conducted under approved animal use protocols from the University of California San Diego Institutional Animal Care and Use Committee (S05480) and in accordance to Antarctic Conservation Act permits provided by the U.S. National Science Foundation to S. Emslie (2006-001) and R. Holt (2008-008).

### Stomach contents, feather and prey samples

Fieldwork took place in January and February of 2008 and 2009 at a colony of approximately 4,500 breeding pairs of Chinstrap penguins and 800 breeding pairs of Gentoo penguins at Cape Shirreff. We collected stomach content samples during the chick-rearing period after chicks had reached the crèche stage (>2.5 weeks of age). We sampled 2–5 unique breeding adults returning from foraging trips between 15:00–17:00 local time at 5 to 7-day intervals for a total of 10–14 Gentoo penguins and 30 Chinstrap penguins each year. We used the water-offloading technique following a modification of the CCAMLR Ecosystem Monitoring Program (CEMP) Standard Methods [37]. Specifically, we did not analyze the complete contents of the stomach; rather we took approximately one-half (about 350 g). Most of the food beneath this upper portion is heavily digested and is difficult to objectively separate by prey species and its inclusion may bias both prey identification and diet composition estimates [10], [38]. We further justify this sampling method as parents ordinarily do not feed their entire food load to the chicks [39], [40]. Excess liquid was removed from each stomach sample by straining it through a fine sieve before weighing to obtain a sample mass (wet weight). From these samples, we determined the percentage of krill, fish, and other material by frequency occurrence and weight. We recovered fish otoliths from diet samples by swirling samples in a dark-bottomed pan and identified otoliths to the lowest possible taxonomic level using an internal reference collection and a published otolith guide [41]. We calculated the frequency occurrence and the minimum number of individuals (MNI) of each fish taxa following standard methods [42]. Specially, we estimated MNI by summing the higher number of either right or left otoliths with half the number of eroded otoliths of unknown side to provide a conservative estimate of the total MNI represented in each stomach sample [42]. In addition, we used otolith measurements and published regression equations to calculate a total and percent of total reconstituted mass for each fish taxa identified (Table S1) [7], [13], [41], [43]. Due to the high number of small *Pleuragramma antarcticum* otoliths recovered, we measured a random sub-sample of 20–75 *P. antarcticum* otoliths per sample and used these values to estimate reconstituted mass for this species.

In February of each year, we collected three breast feathers from a random sample of 18–20 fledgling chicks of each species while they were preparing to leave their natal colonies for the sea at 7–10 weeks of age. From 2005 to 2009, we collected representative samples of penguin prey species during trawls conducted along the South Shetland Islands and northern Antarctic Peninsula and kept samples frozen prior to analysis. We further supplemented this prey library with published isotopic values of two fish prey, *Protomyctophum bolini* and *Champsocephalus gunnari* [44], [45].

### Stable isotope analysis

We cleaned feathers using a 2∶1 chloroform ∶ methanol rinse, air-dried and cut them into small fragments with stainless steel scissors. We homogenized whole prey samples, dried them for 48 hours in an oven at 60°C and then extracted lipids from these samples using a Soxhlet apparatus with a 1∶1 Petroleum-Ether: Ethyl-Ether solvent mixture for 8 hours [46]. We flash-combusted (Costech ECS4010 elemental analyzer) approximately 0.5 mg of each feather and prey sample loaded into tin cups and analyzed for carbon and nitrogen isotopes (δ^13^C and δ^15^N) through an interfaced Thermo Delta V Plus continuous flow stable isotope ratio mass spectrometer (CFIRMS). Raw δ values were normalized on a two-point scale using glutamic acid reference materials with low and high values (i.e. USGS-40 (δ^13^C = −26.4‰, δ^15^N = −4.5‰) and USGS-41 (δ^13^C = 37.6‰, δ^15^N = 47.6‰)). Sample precision based on repeated sample and reference material was 0.1‰ and 0.2‰, for δ^13^C, and δ^15^N, respectively.

Stable isotope ratios are expressed in δ notation in per mil units (‰), according to the following equation:
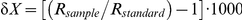
Where X is ^13^C or ^15^N and R is the corresponding ratio ^13^C/^12^C or ^15^N/^14^N. The *R*_standard_ values were based on the Vienna PeeDee Belemnite (VPDB) for δ^13^C and atmospheric N_2_ for δ^15^N.

### Isotopic mixing models

We used four model variants of the SIAR Bayesian mixing model [21] in the R environment (R Development Core Team 2007) to explore our ability to quantify chick diet composition (Table S2). The SIAR model estimates probability distributions of multiple source contributions to a mixture while accounting for the observed variability in source and mixture isotopic signatures, dietary isotopic fractionation, and elemental concentration. We used two SIAR model variants with two prey sources (Antarctic krill vs. “fish”) to estimate diet composition for each species/year combination using the δ^13^C and δ^15^N values of chick feathers. Model 1 uses the δ^13^C and δ^15^N values of a representative fish species, *P. antarcticum*, which is commonly found in *Pygoscelis* penguin diets as the “fish” source [6]. Model 2 uses species and year specific “fish” δ^13^C and δ^15^N values calculated by averaging the δ^13^C and δ^15^N values of multiple fish species weighted by their relative percent reconstituted fish mass (Tables S1 and S2).

We used two additional variants of the SIAR mixing model with multiple prey sources (6–7 depending on penguins species) to further evaluate methods of integrating stomach content data to better elucidate the taxonomic composition of the fish portion of penguin diets. For these models, we restricted our analyses to chick feather data from 2008 when the fish portion of chick diets was the most diverse. Model 3 is an initial multi-source model estimating the relative contribution of krill (*E. superba*) and all fish species in our prey library identified from otoliths in each species' stomach contents (Tables S1 and S2). Model 4 is an *a posteriori* informed model where we restricted the resulting posterior draws to those in which the relative importance of individual fish species was ranked in accordance to the abundance of each species identified through otolith analysis. For Model 4, we restricted posterior draws to only those where the estimated proportional contributions of the most abundant fish prey based on reconstituted fish mass was greater than the estimated proportional contributions of the second most abundant fish prey, and for the second most abundant fish prey greater than the third most abundant and so on for all fish species. For both the initial (Model 3) and informed (Model 4) multi-source models, we also summed results across fish prey and estimated the proportional contribution of each fish species to the fish portion (i.e. excluding krill) of penguin diets. For all SIAR models we incorporated *Pygoscelis* penguin feather δ^15^N and δ^13^C discrimination factors [47] and ran 1 million iterations, thinned by 15, with an initial discard of the first 40,000 resulting in 64,000 posterior draws.

### Statistical analysis

Statistical calculations were performed using SAS (Version 9.1). We analyzed SCA data to test for differences between years and species using separate generalized linear models (Proc Genmod). We used a binomial error distribution and logit link function for generalized linear models with the percent composition (by wet mass) or frequency occurrence of each of our three main prey group (krill, fish, and ‘other’ prey) as the response variables. For models that used MNI of fish and reconstituted fish mass per sample as the response variables, we used a Poisson-error distribution with a logit link function. For all generalized linear models we conducted post-hoc analyses using a Bonferroni correction and reported chi-square and p-values from the likelihood ratio test statistics for type 3 tests.

To test for differences in the chick feather δ^13^C and δ^15^N values we used multivariate analysis of variance (MANOVA) along with Tukey-Kramer Multiple comparison values across species and years using PROC ANOVA. We used a similar MANOVA to examine the δ^13^C and δ^15^N values of species in our prey library. We used model 95% credibility intervals to compare estimates of krill vs. fish among two-source SIAR model variants (Models 1 and 2) and SCA wet mass, and the percent contribution of individual fish species to fish portion of chick diets among multi-source SIAR model variants (Models 3 and 4) and SCA otolith-derived reconstituted fish mass. To facilitate direct comparison between SIAR models and SCA, we calculated Bayesian averages and 95% credibility intervals for each SCA dataset using Markov chain Monte Carlo (MCMC) simulations via WinBUGS (Version 1.4). These MCMC simulations were implemented using the non-informative Dirichlet prior with an identical number of iterations, thins, and discards as our SIAR model analysis. Furthermore, we used Chi-Square goodness of fit tests to compare the distribution of mean estimates of the percent contribution of individual prey fish species to diets among multi-source SIAR models variants and SCA data.

Data were examined for normality and equal variance, all tests were two-tailed and significance was assumed at the 0.05 level. Stable isotope values of chick feathers and prey species are presented ± standard deviation (SD), while diet composition estimates from stomach content analysis are presented ± standard error (SE) in tables and ±95% credibility intervals in figures.

## Results

### Stomach content analysis

Chinstrap penguin stomach samples had a higher percent contribution of krill relative to Gentoo penguin samples (Table 1a; χ^2^_1_ = 10.91, p = 0.0010). However, we found no differences by year (χ^2^_1_ = 0.22, p = 0.6375) or a species*year interaction (χ^2^_1_ = 0.00, p = 0.9805). Similarly, Gentoo penguin samples contained a significantly higher percent contribution of fish relative to Chinstrap penguin samples, (χ^2^_1_ = 12.24, p = 0.0005), but we could not detect differences across years (χ^2^_1_ = 0.08, p = 0.7755) or a species*year interaction (χ^2^_1_ = 0.26, p = 0.6078). The percent contribution to stomach samples of other prey species, including cephalopods, amphipods and other euphausiid species did not differ by penguin species (χ^2^_1_ = 0.00, p = 0.9694), year (χ^2^_1_ = 0.36, p = 0.5468) or a species*year interaction (χ^2^_1_ = 0.00, p = 0.9694).

**Table 1:** The composition and occurrence of common prey groups and the minimum number of individual fish and reconstituted fish mass recovered from penguin stomach contents.

		a) Percent composition of stomach contents by wet mass (% FO)			b) Fish content per stomach sample based on otoliths (total)	
Species, year	*n*	Krill - *E. superba*	Fish	Other	MNI	Reconstituted mass (g)
Chinstrap penguin						
2008	30	99.6±0.3^a^	0.4±0.3^b^	0.0±0.0^a^	1.8±0.7^a^	31.2±15.7^a^
		(100.0)	(36.7)	(10.0)	(65)	(936.3)
2009	30	99.1±0.9^a^	0.0±0.0^a^	0.9±0.9^a^	1.4±0.4^a^	3.4±0.9^b^
		(100.0)	(50.0)	(10.0)	(45)	(103.0)
Gentoo penguin						
2008	10	83.7±9.6^a^	16.3±9.6^b^	0.0±0.0^a^	10.9±4.3^b^	155.5±43.1^c^
		(90.0)	(100.0)	(20.0)	(109)	(1555.5)
2009	14	68.2±10.8^a^	30.8±10.4^b^	0.9±0.7^a^	211.9±70.5^c^	294.3±80.4^d^
		(100.0)	(100.0)	(21.4)	(2967)	(4119.6)

Other prey include cephalopods, Hyperiid amphipods, and small euphausiids (primarily *Thysanoessa macrura*). Groups that do not share at least one superscript within a column are significantly different for the variable in question at the 0.05 level. Values are presented mean ± SE, with the frequency of occurrence (% FO) of common prey species and the total minimum number of individual (MNI) fish and reconstituted fish mass in grams presented in parentheses.

We found evidence of krill in all Chinstrap penguin samples and in all but one Gentoo penguin sample (Table 1a). We found evidence of fish in all Gentoo penguin samples and in 36.7–50.0% of Chinstrap penguin samples, even when there was no detectable wet mass of fish (Table 1a). However, the frequency occurrence of fish in Chinstrap penguin samples did not differ across years (χ^2^_1_ = 1.09, p = 0.2966). Similarly, the frequency occurrence of other prey species did not differ by penguin species (χ^2^_1_ = 1.57, p = 0.2107), year (χ^2^_1_ = 0.00, p = 0.9481), or a species*year interaction (χ^2^_1_ = 0.00, p = 0.9481).

The MNI of fish and reconstituted fish mass per sample differed between species and years (Table 1b). Chinstrap penguin diets had lower MNI and reconstituted fish mass than Gentoo penguins (MNI: χ^2^_1_ = 959.14, p<0.0001; reconstituted mass: χ^2^_1_ = 959.14, p<0.0001). Across species and years Chinstrap penguins had higher reconstituted fish mass in diet samples in 2008 relative to 2009, while Gentoo penguins had both lower MNI and reconstituted fish masses in 2006 relative to 2009 (MNI: χ^2^_1_ = 147.74, p<0.0001; reconstituted mass: χ^2^_1_ = 1122.46, p<0.0001). A total of 96.3% of all otoliths were identifiable to at least the genus level, with six and five fish taxa represented in Chinstrap and Gentoo penguin diets samples, respectively (Table S1).

### Isotopic signatures of chick feathers and prey

**Figure 1:**
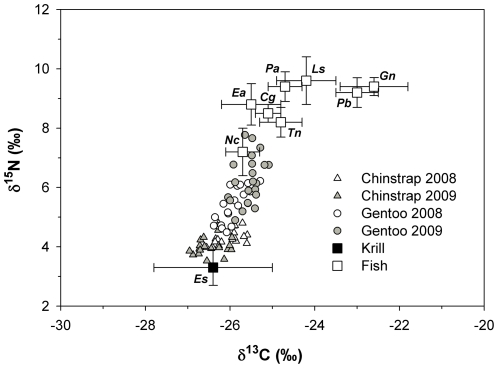
Isotope signatures of penguin chick feathers in relation to nine common prey species. Values are presented (δ^13^C and δ^15^N; mean ± SD). Chick feather values are presented after correction for dietary isotopic discrimination (Polito et al. 2011). Prey species abbreviation are Krill: Es (*Euphausia superba*), Fish: Ea (*Electrona antarctica*), Cg (*Champsocephalus gunnari*), Gn (*Gymnoscopelus nicholsi*), Ls (*Lepidonotothen squamifroms*), Nc (*Notolepis coatsi*), Pa (*Pleuragramma antarcticum*), Pb (*Protomyctophum bolini*), and Tn (*Trematomus newnesi*).

We found δ^15^N and δ^13^C values of penguin chick feathers differed by species (Wilks' λ, p<0.0001), year (Wilks' λ, p = 0.0409) and had a significant species*year interaction (Wilks' λ, p<0.0001). Gentoo penguin chicks had higher feather δ^15^N values than Chinstrap penguin chicks in both years (Table 2, Fig. 1). However, while Gentoo penguin chick feather δ^15^N values were higher in 2009 relative to 2008, Chinstrap penguin chick feather δ^15^N values did not differ between years. Chinstrap and Gentoo penguin chicks had similar feather δ^13^C values in 2008, but lower and higher values for Chinstrap and Gentoo penguins in 2009, respectively (Table 2). We found δ^15^N and δ^13^C values of species in our library of common penguin prey items also differed significantly (Wilks' λ, p<0.0001). The δ^15^N and δ^13^C values differed greatly between krill and fish species, while isotope values overlapped among many fish species (Table 2, Fig. 1).

**Table 2:** The carbon to nitrogen ratio and stable isotope signatures of penguin chick feathers and nine common krill and fish prey species.

Group, taxa or year	*n*	C/N	δ^15^N (‰)	δ^13^C (‰)
Chick feathers				
Chinstrap penguin, 2008	20	3.1±0.1	7.8±0.3^a^	−24.7±0.3^a^
Chinstrap penguin, 2009	20	3.1±0.1	7.5±0.3^a^	−25.2±0.3^b^
Gentoo penguin, 2008	20	3.1±0.1	8.9±0.6^b^	−24.6±0.3^a^
Gentoo penguin, 2009	21	3.1±0.1	9.8±0.8^c^	−24.3±0.3^c^
Prey library				
Krill, *Euphausia superba*	40	3.7±0.2	3.3±0.6^a^	−26.4±1.4^a^
Fish, *Protomyctophum bolini*	13	3.2±0.1	9.2±0.5	−23.0±0.5
Fish, *Electrona antarctica*	41	3.3±0.1	8.8±0.7^b^	−25.5±0.7^b^
Fish, *Gymnoscopelus nicholsi*	6	3.4±0.1	9.4±0.3^bc^	−22.6±0.8^c^
Fish, *Notolepis coatsi*	3	3.2±0.1	7.2±0.8^d^	−25.7±0.4^abd^
Fish, *Lepidonotothen squamifroms*	10	3.3±0.1	9.6±0.8^c^	−24.2±0.7^d^
Fish, *Pleuragramma antarcticum*	30	3.4±0.2	9.4±0.5^c^	−24.7±0.4^d^
Fish, *Trematomus newnesi*	10	3.3±0.1	8.2±0.5^bd^	−24.8±0.5^bd^
Fish, *Champsocephalus gunnari*	5	3.3±0.1	8.5±0.3	−25.1±0.3

Carbon to nitrogen ratios (C/N) and stable isotope values (δ^15^N & δ^13^C) are presented mean ± SD. Chick feathers and prey species that do not share at least one superscript within a column for each group (feathers or prey) are significantly different for the variable in question at the 0.05 level. *P. bolini* [45] and *C. gunnari* [44] were not included in prey species analyses.

### Two-source SIAR models

**Figure 2:**
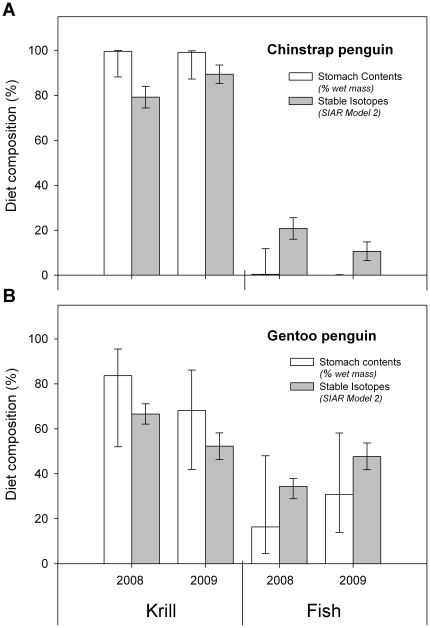
The estimated diet composition of penguin chicks based on stomach content and stable isotope analysis. Stomach content proportions are calculated as a percent of wet mass and proportion estimates of krill vs. fish using stable-isotope analysis are derived from a two-source Bayesian mixing model SIAR (Model 2) using annually weighted “fish” values listed in table S2 [21]. Proportions are presented mean ± Bayesian 95% credibility intervals.

The two-source SIAR model variant that used *P. antarcticum* isotopic values as a representative “fish” source (Model 1) and the variant that used a year and species-specific weighted “fish” isotopic values (Model 2) both predicted that Gentoo penguin chicks consumed relatively less krill and more fish than Chinstrap penguins in both years (Table 3). However, when examining model 95% credibility intervals these two model variants differed in their ability to detect species-specific, inter-annual differences in diet composition. While both two-source SIAR model variants predicted that Gentoo penguin chick diets contained a higher percentage of krill in 2008, only Model 2 detected a larger amount of fish in Chinstrap penguin chick diets during 2008 relative to 2009 (Table 3). Two-source SIAR model variants predicted a higher contribution of fish in the chick diets of both penguin species in comparison to diet composition estimates derived from SCA wet mass (Fig. 2). SCA estimates were also more variable than SIAR model predictions for Gentoo penguin chick diets. Furthermore, SCA derived estimates of the mean contribution of krill and fish in both species' diets fell outside of our two-source SIAR models 95% upper and low credibility intervals, respectively (Fig. 2; Tables 1 and 3).

**Table 3:** Predicted diet composition of penguin chicks at Cape Shirreff, Livingston Island derived from stable isotope analysis using two variants of the SIAR two-source Bayesian mixing model.

	SIAR δ^15^N & δ^13^C two source models			
	Model 1: *P. antarcticum*		Model 2: weighted by % mass	
Species, year	% Krill	% Fish	% Krill	% Fish
Chinstrap penguin				
2008	83.8 (80.1–87.7)	16.2 (12.3–19.9)	79.2 (74.4–84.0)	20.8 (16.0–25.6)
2009	89.4 (85.2–93.5)	10.6 (6.5–14.8)	89.4 (85.2–93.5)	10.6 (6.5–14.8)
Gentoo penguin				
2008	69.1 (64.9–73.2)	30.9 (26.8–35.1)	66.6 (62.1–71.1)	34.4 (28.9–37.9)
2009	53.1 (47.1–58.9)	46.9 (41.1–52.9)	52.3 (46.3–58.2)	47.7 (41.8–53.7)

Diet compositions were estimated using SIAR [21] and are presented as mean estimates with 95% credibility intervals (in parentheses). Model 1 uses the δ^15^N and δ^13^C values of a representative fish species, *Pleuragramma antarcticum*, as the ‘fish’ source while Model 2 use yearly and species-specific weighted ‘fish’ δ^15^N and δ^13^C values (Table S1 and S2).

### Multi-source SIAR models

**Figure 3:**
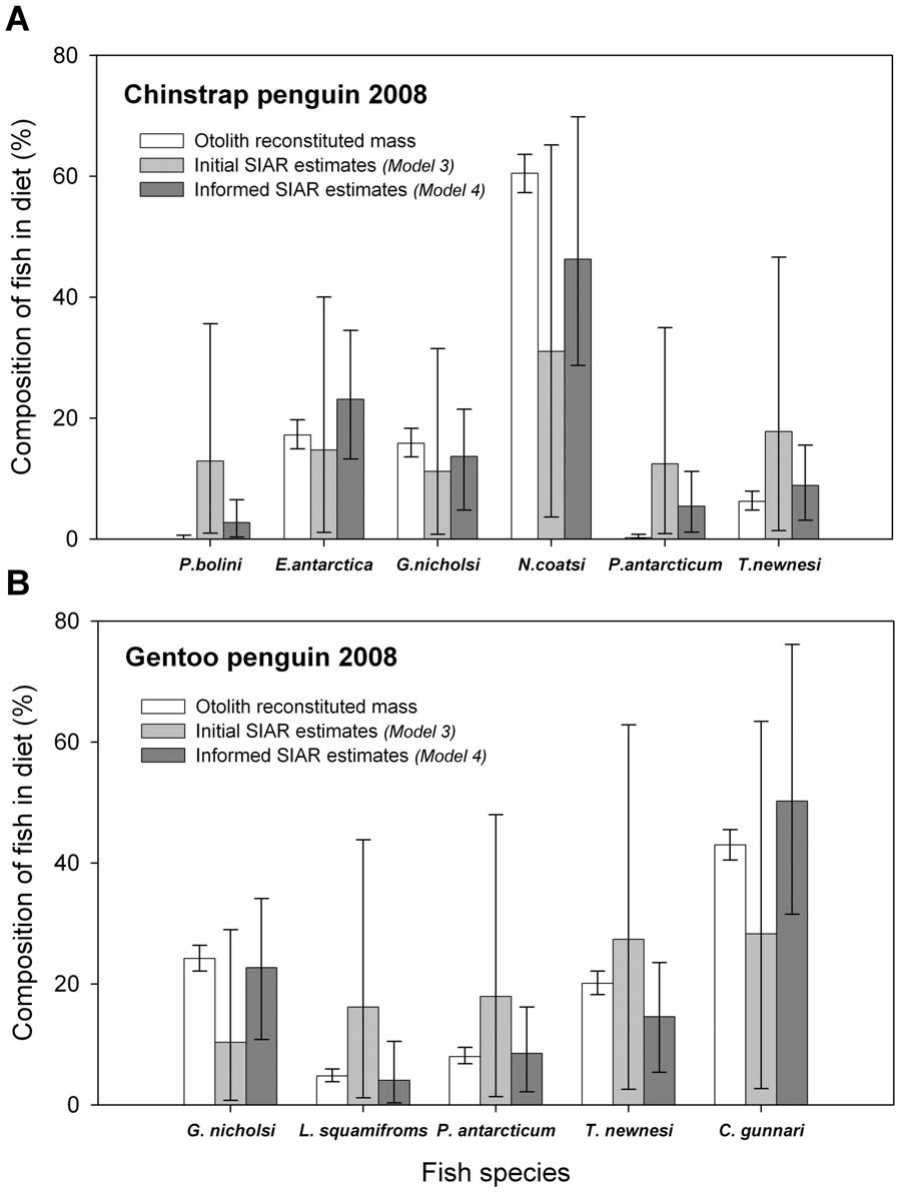
The fish species composition of penguin chick diets based on otolith and stable isotope analysis. Estimated dietary contributions exclude the krill portion of chick diets. Reconstituted mass derived from otolith measurements are compared with two variants (Models 3 and 4) of the SIAR multi-source Bayesian mixing model [21]. An initial model estimating the relative contribution of individual fish species identified from otoliths in stomach contents and an a posteriori informed model restricted to posterior draws agreeing with the relative abundance of each fish species by reconstituted mass (Tables S1 & S2). Estimates are presented mean ± Bayesian 95% credibility intervals.

Both multi-source SIAR model variants (Models 3 and 4) predicted that Antarctic krill comprised the largest prey component of Chinstrap and Gentoo penguin chick diets in 2008 (Table 4). In addition both multi-source SIAR models broadly agreed with two-source SIAR model estimates of the relative proportion of krill vs. all fish species summed (Tables 3 and 4). However, our initial multi-source SIAR model (Model 3) had difficulty estimating the relative proportion of individual fish species to both penguin species chick diets in 2008. SIAR Model 3's 95% credibility intervals broadly overlapped across fish species and the mean relative proportion of each fish species differed from estimates using otolith reconstituted mass (Table 4, Fig. 3; Chinstrap: χ^2^_5_ = 62.65, p<0.0001; Gentoo: χ^2^_4_ = 41.70, p<0.0001).

**Table 4:** Predicted diet compositions of penguin chicks at Cape Shirreff, Livingston Island derived from stable isotope analysis using two variants of a multi-source Bayesian mixing model.

	SIAR δ^15^N & δ^13^C multi source models			
	Chinstrap 2008		Gentoo 2008	
Prey source	Initial model	Informed model	Initial model	Informed model
Krill				
*Euphausia superba*	79.4 (74.4–84.2)	78.1 (73.5–81.6)	65.2 (59.6–70.6)	65.2 (61–69.1)
Fish				
*Protomyctophum bolini*	2.6 (0.0–7.0)	0.6 (0.1–1.4)	-	-
*Electrona antarctica*	3.0 (0.0–8.0)	5.0 (2.7–8.3)	-	-
*Gymnoscopelus nicholsi*	2.2 (0.0–6.1)	2.9 (1.1–4.6)	3.5 (0.0–9.6)	7.8 (3.8–11.8)
*Notolepis coatsi*	6.7 (0.0–15.3)	10.3 (5.7–16.5)	-	-
*Lepidonotothen squamifroms*	-	-	5.5 (0.0–14.8)	1.4 (0.1–3.6)
*Pleuragramma antarcticum*	2.5 (0.0–6.9)	1.2 (0.3–2.4)	6.1 (0.0–16)	2.9 (0.8–5.4)
*Trematomus newnesi*	3.6 (0.0–9.5)	1.9 (0.7–3.3)	9.6 (0.0–22.8)	5.0 (1.9–8.3)
*Champsocephalus gunnari*	-	-	10 (0.0–23)	17.6 (10.2–27.7)
All Fish	20.6 (15.8–25.6)	21.9 (18.4–26.5)	34.8 (29.4–40.4)	34.8 (30.9–39)

Diet compositions were estimated using SIAR [21] and are presented as mean estimates with 95% credibility intervals (in parentheses). The initial model (SIAR Model 3) estimates the relative contribution of individual krill and fish species identified in stomach contents to overall penguin diets. The informed model (SIAR Model 4) restricts posterior draws of diet composition estimates to those agreeing with the relative abundance of each fish species based on reconstituted mass (Tables S1 & S2). All fish represents the sum of the predicted contribution of all fish species.

In contrast, the *a posteriori* informed multi-source SIAR model (Model 4) performed better than the initial multi-source SIAR model (Model 3) at estimating the species composition of the fish portion of chick diets. While Model 4's prediction of the mean relative proportion of each fish species in Chinstrap penguin chick diets differed slightly from estimates from otolith reconstituted mass (χ^2^_5_ = 14.55, p = 0.0125), the resulting 95% credibility intervals were reduced by 53.5±17.2% in comparison to Model 3 (range: 33.2–82.2%; Table 4, Fig. 3). Furthermore, Model 4 prediction's of the mean relative proportion of each fish species in Gentoo penguin chicks' diets was similar to estimates from otolith reconstituted mass (χ^2^_4_ = 3.40, p = 0.4949). In addition, the resulting 95% credibility intervals were reduced by 52.0±27.7% in comparison to Model 3 (range: 17.4–76.2%; Table 4, Fig. 3).

## Discussion

### Stomach content analysis

Our SCA analysis highlights several of the possible biases inherent when using this method. Similar to previous studies at Cape Shirreff, we observed evidence of fish such as otoliths, scales, and lenses in many Chinstrap penguin samples even when there was no measurable amount of fish tissue by wet mass [34], [36]. This evidence suggests that fish biomass consumed by adults digests completely prior to their return to the breeding colony or, more likely, is delivered to chicks in the heavily-digested component of adult stomach contents which cannot be objectively quantified [6], [10]. Furthermore, because we collected stomach samples during the late afternoon, our sample does not include adults who foraged at night and tend to have a much higher percentage and occurrence of fish in their stomach samples [34], [48]. In addition, diet composition estimates derived from SCA in our study were often highly variable, making it difficult to detect differences among years and penguin species (Table 1). This finding does not appear to be unique in seabird dietary studies using SCA, which often requires high sample sizes and large differences between groups to detect inter-annual or species-specific differences in diet composition [3], [9]. However, our study suggests that the analysis of otoliths can still provide detailed information on species-specific and temporal variation in the consumption of fish prey species when overall diet composition estimates derived from stomach content wet mass are less informative.

### Two-source, SIAR models

Two-source SIAR models predicted a relatively greater contribution of fish to chick-rearing diets in both species in comparison to SCA biomass estimates. This result is not unexpected as SCA is thought to underestimate the amount of fish in these species' diets due to the digestion and diel biases described above [10], [12], [48]. In addition, two-source SIAR models also provided the least variable predictions of diet composition in comparison to SCA. The SIA of chick feathers provided an average value of each individual chick's diet throughout the time of feather growth during the chick-rearing period [18], [19]. In contrast, SCA data represent a series of “snap-shots” (in this study every 5 to 7 days) of the food that one of two parents feed its chick [3]. Our study suggests that SIA of tissues that integrate diets over long time periods are innately less variable than SCA given a similar sample size and are more appropriate for examining inter-annual differences in chick diets. For example, the two-source SIAR models used in our study were able to identify inter-annual and species-specific differences in the relative abundance of fish and krill in diets not readily apparent using SCA.

When prior information on prey species composition is limited, such as outside the breeding season, using a representative prey source in isotopic mixing models can provide important information on seabird diets when little else is known [24]. However, our results also suggest that variation in prey species composition within trophic or functional groups can mask significant differences in diet composition that would not be apparent from isotopic values or mixing model predictions using only representative prey sources. This result was most apparent when examining the effect of fish prey δ^15^N values on chick feather δ^15^N values and the two-source isotopic mixing models used in our study. For example, Chinstrap penguin chick feather δ^15^N values did not differ between years (Table 2). In addition, the 95% credibility intervals of dietary estimate from the two-source SIAR model using *P. antarcticum* as the fish prey source (Model 1) overlapped between years (Tables 3). In contrast, 95% credibility intervals of two-source SIAR model using yearly and species-specific weighted “fish” values (Model 2) suggest a greater abundance of fish in Chinstrap penguin chick diets in 2008 relative to 2009, which was confirmed by otolith derived, average reconstituted fish mass. In 2008, the fish portion of Chinstrap penguin chick diets was composed of six fish species with an estimated δ^15^N value of 7.9±0.7‰, while *P. antarcticum* (δ^15^N: 9.4±0.5‰) was the only fish species in 2009 diets (Tables S1 and S2). While this 1.5‰ difference is small relative to 4.6–6.1‰ differences between fish and krill, it was enough to confound inter-annual comparisons of Chinstrap penguin chick diets in our study.

### Multiple-source, SIAR models

When parameterizing our two multi-source SIAR models we used otolith data to select the appropriate fish prey sources to include in each species model (Tables S1 and S2). However, our initial multi-source SIAR model (Model 3) had difficulty precisely estimating the individual species composition of the fish portion of penguin diets due to the general similarities in δ^13^C and δ^15^N values among many of the fish species included as prey sources (Fig. 3). Antarctic fish species generally consume krill and other fish species and due to their similar tropic level, these fish species tend to have similar δ^15^N values [45]. While variation in the δ^13^C values of Antarctic fish species occupying different habitats can occur, overlap among the isotopic values of fish within the prey-size range of penguins is common [45]. In addition, the δ^13^C values of marine organisms can be affected by factors other than diet and habitat such as seasonal variations in primary production [49]. These issues can confound the use of isotopic models when estimating the relative contribution to predator diets of individual prey species occupying similar trophic levels such as fish.

We found that using SCA data to *a posteriori* refine multi-source SIAR model outputs (Model 4) can provide greater resolution when estimating the contributions of isotopically similar prey species. When reducing our multi-source SIAR model's posterior predictions to only those outcomes in which the importance of individual fish species were ranked similarly to estimates from otolith data, our informed multi-source SIAR model (Model 4) provided mean relative diet contributions that generally agreed with reconstituted fish masses and greatly reduced 95% credibility intervals relative to our initial SIAR multi-source model (Table 4, Fig. 3). Although not used in this study, the SIAR model package also allows users to input *a priori* estimates of the relative contribution of each prey species [21]. Informing our multi-source SIAR models in this manner would have required us to provide accurate estimates of the contribution of Antarctic krill as well as each fish species to penguin diets. However, by using this method, any biases from both SCA biomass and otolith data would be incorporated into the model predictions. In contrast, we used a simple *a posteriori* ranking method that, while fitting fish prey species to data derived from otoliths, provided no assumptions about the relative contribution of krill to penguin diets. Therefore, unlike *a priori* estimates, our method put no constraints on the relative abundance of krill vs. all fish species combined while still reducing the 95% credibility intervals by approximately one-half relative to initial models.

### Integrating SCA and SIA when estimating seabird diets

Our findings suggest that SIA can have greater accuracy than SCA to track inter-annual and species-specific variations in diet composition at broad trophic levels (i.e. zooplankton vs. fish). By focusing on tissues that integrate diets over long periods of time, SIA can avoid many of the digestive and temporal biases of SCA and provide less variable estimates of seabird diets in a less invasive manner. Therefore, when prey items identified from previous studies are isotopically distinct or can be combined into biologically meaningful groups, SIA alone may be sufficient to address a particular question without the need for additional SCA.

In contrast, it appears difficult to use SIA methods to estimate the fine scale taxonomic composition of seabird diets to the same degree as is generally possible through SCA. However, we found that when this level of accuracy is required, it is possible to integrate these two methods to produce more refined estimates of diets. Simultaneously conducted SCA data can be used to weight *a priori* combinations of isotopically similar prey in two-source mixing models to better predict diets at broad trophic levels. In addition, when using multi-source models, SCA can first inform which prey sources should be incorporated into models and second, *a posteriori* refined model predictions of prey contributions to better track inter-annual and species-specific differences in seabird diets using SIA. Moreover, as with all studies estimating diets using SIA, it is important to use taxonomically appropriate discrimination factors in isotopic mixing models, as they can be sensitive to these values [24], [50].

## Supporting Information

Table S1 **The composition and reconstituted mass of fish species identified from otoliths in Chinstrap and Gentoo penguin stomach contents.**
(PDF)

Table S2 **Discrimination factors, sample sizes, isotope values and elemental concentrations of prey source inputs into SIAR mixing models.**
(PDF)
