# Supplementary material for: Integrating Stomach Content and Stable Isotope Analyses to Quantify the Diets of Pygoscelid Penguins
Source: PLoS One. 2011 Oct 28;6(10):e26642. doi: 10.1371/journal.pone.0026642 (PMC3203888; doi:10.1371/journal.pone.0026642)
Supplement: Table S1 — The composition and reconstituted mass of fish species identified from otoliths in Chinstrap and Gentoo penguin stomach contents. (PDF) [file pone.0026642.s001.pdf]

Table S1. The frequency occurrence (%FO), minimum number of individuals (MNI), percent of MNI (%MNI), reconstituted mass (Mass), and percent of reconstituted mass (%Mass) of fish species identified from stomach contents collected from adult Chinstrap and Gentoo penguins during the crèche period at Cape Shirreff, Livingston Island in 2008 and 2009.

	2008					2009				
Penguin species, fish prey	%FO	MNI	%MNI	Mass (g)	%Mass	%FO	MNI	%MNI	Mass (g)	%Mass
Chinstrap penguin										
Protomyctophum bolini	3.3	1	1.9	0.5	0.1	0.0	0	0.0	0.0	0.0
Electrona antarctica	23.3	28	52.8	161.1	17.2	0.0	0	0.0	0.0	0.0
Gymnoscopelus nicholsi	13.3	5	9.4	148.4	15.8	0.0	0	0.0	0.0	0.0
Notolepis coatsi	13.3	14	26.4	566.5	60.5	0.0	0	0.0	0.0	0.0
Pleuragramma antarcticum	3.3	2	3.8	1.9	0.2	43.3	42	100.0	103.0	100.0
Trematomus newnesi	10.0	3	5.7	58.0	6.2	0.0	0	0.0	0.0	0.0
Gentoo penguin										
Gymnoscopelus nicholsi	30.0	11	10.1	375.7	24.2	0.0	0	0.0	0.0	0.0
Lepidonotothen sp.	40.0	13	11.9	74.4	4.8	42.9	13	0.4	41.2	1.0
Pleuragramma antarcticum	40.0	61	56.0	124.8	8.0	92.9	2947	99.3	3701.3	89.8
Trematomus newnesi	40.0	4	3.7	312.2	20.1	7.1	1	0.0	179.6	4.4
Champsocephalus gunnari	50.0	14	12.8	668.4	43.0	21.4	4	0.1	197.6	4.8
Unknown fish sp.	50.0	6	5.5	-	-	7.1	2	0.1	-	-
