# Supplementary material for: Integrating Stomach Content and Stable Isotope Analyses to Quantify the Diets of Pygoscelid Penguins
Source: PLoS One. 2011 Oct 28;6(10):e26642. doi: 10.1371/journal.pone.0026642 (PMC3203888; doi:10.1371/journal.pone.0026642)
Supplement: Table S2 — Discrimination factors, sample sizes, isotope values and elemental concentrations of prey source inputs into SIAR mixing models. (PDF) [file pone.0026642.s002.pdf]

Table S2. Discrimination factors, sample sizes, isotopic signatures and elemental concentrations of prey source inputs into SIAR mixing models [21].

**Model, Inputs**	***n***	**δ^{15}N (‰)**	**δ^{13}C (‰)**	**% N**	**% C**	**Source**
SIAR Model 1						
Discrimination factor	-	3.5±0.4	1.3±0.5	-	-	[47]
Source 1: Krill (*E. superba*)	40	3.3±0.6	-26.4±1.4	10.0±1.0	37.0±4.2	-
Source 2: Fish (*P. antarcticum*)	30	9.4±0.5	-24.7±0.4	12.0±1.0	40.0±2.0	[24]
SIAR Model 2						
Discrimination factor	-	3.5±0.4	1.3±0.5	-	-	[47]
Source 1: Krill (*E. superba*)	40	3.3±0.6	-26.4±1.4	10.0±1.0	37.0±4.2	-
Source 2: Fish (Weighted by %mass)						
Chinstrap 2008	11	7.9±0.7	-25.1±0.5	12.8±1.0	41.0±1.2	-
Chinstrap 2009	30	9.4±0.5	-24.7±0.4	12.0±1.0	40.0±2.0	-
Gentoo 2008	8	8.8±0.4	-24.4±0.5	12.4±1.0	40.8±1.8	-
Gentoo 2009	28	9.3±0.5	-24.7±0.4	12.0±1.0	40.0±2.0	-
SIAR Models 3 & 4 (2008 only)						
Chinstrap Penguins:						
Discrimination factor	-	3.5±0.4	1.3±0.5	-	-	[47]
Source 1: Krill (*E. superba*)	40	3.3±0.6	-26.4±1.4	10.0±1.0	37.0±4.2	-
Source 2: Fish (*P. bolini*)	13	9.2±0.5	-23.0±0.5	12.0±1.0[1]	40.0±2.0[1]	[45]
Source 3: Fish (*E. antarctica*)	41	8.8±0.7	-25.5±0.7	12.0±1.0	39.0±2.0	-
Source 4: Fish (G. *nicholsi*)	6	9.4±0.3	-22.6±0.8	13.0±1.0	43.0±1.0	-
Source 5: Fish (*N. coatsi*)	3	7.2±0.8	-25.7±0.4	13.0±1.0	41.0±1.0	-
Source 6: Fish (*P. antarcticum*)	30	9.4±0.5	-24.7±0.4	12.0±1.0	40.0±2.0	[24]
Source 7: Fish (*T. newnesi*)	10	8.2±0.5	-24.8±0.5	13.0±1.0	41.0±2.0	-
Gentoo Penguins:						
Discrimination factor	-	3.5±0.4	1.3±0.5	-	-	[47]
Source 1: Krill (*E. superba*)	40	3.3±0.6	-26.4±1.4	10.0±1.0	37.0±4.2	-
Source 2: Fish (*G. nicholsi*)	6	9.4±0.3	-22.6±0.8	13.0±1.0	43.0±1.0	-
Source 3: Fish (*L. squamifroms*)	10	9.6±0.8	-24.2±0.7	11.0±1.0	37.0±2.0	[24]
Source 4: Fish (*P. antarcticum*)	30	9.4±0.5	-24.7±0.4	12.0±1.0	40.0±2.0	[24]
Source 5: Fish (*T. newnesi*)	10	8.2±0.5	-24.8±0.5	13.0±1.0	41.0±2.0	-
Source 6: Fish (*C. gunnari*)	5	8.5±0.3	-25.1±0.3	12.0±1.0	40.0±2.0	[44]

%N and %C values for *P. bolini* and *C. gunnari* were not available so averaged values from the six other fish species examined in this study were used. See respective numbers in reference section of the text for source information.
